# Lessons Learned From the Integration of Ambient Assisted Living Technologies in Older Adults’ Care: Longitudinal Mixed Methods Study

**DOI:** 10.2196/57989

**Published:** 2025-06-11

**Authors:** Oteng Ntsweng, Martin Kodyš, Zhi Quan Ong, Fang Zhou, Antoine de Marassé-Enouf, Ibrahim Sadek, Hamdi Aloulou, Sharon Swee-Lin Tan, Mounir Mokhtari

**Affiliations:** 1 W.P Carey School of Business, Arizona State University Tempe, AZ United States; 2 Institut Mines-Télécom Paris France; 3 Department of Information Systems and Analytics, National University of Singapore Singapore Singapore; 4 Biomedical Engineering Department, Faculty of Engineering, Helwan University Helwan Egypt; 5 University of Sfax Sfax Tunisia

**Keywords:** ambient assisted living, Internet of Things, IoT, normalization process theory, older adults, quality of life

## Abstract

**Background:**

COVID-19 has given impetus to an already growing trend around the use of ambient assisted living (AAL) technologies to support frail older adults who live alone. However, the challenge is that systematic research on the long-term use of AAL technologies remains in its nascent stages, leaving gaps in the understanding of the predictors that contribute to the routine embedding of AAL technologies in older adults’ care.

**Objective:**

This paper aims to share key lessons from a longitudinal study on the routine embedding of AAL technologies in older adults’ care within a hitherto under-studied Southeast Asian context. Our objective was to design and deploy an AAL system termed Ubiquitous Service Management and Reasoning Architecture (Ubismart), evaluate its impact on older adults’ quality of life (QOL), and distill lessons to inform the sustainable and culturally sensitive adoption of AAL technologies in similar settings.

**Methods:**

We conducted an in-depth case study using a mixed methods design. First, we designed and deployed Ubismart to unobtrusively monitor and visualize older adults’ activities of daily living. To assess changes in QOL, we administered a simplified, gamified version of the validated Older People’s Quality of Life Questionnaire. Finally, we conducted semistructured interviews with older adults and their caregivers to triangulate the quantitative findings and explore evolving perceptions of the technology and its integration into daily routines.

**Results:**

Quantitative analysis revealed significant improvements in (1) psychological and emotional well-being (*P*=.01) and (2) leisure and social activities (*P*=.03) following the AAL intervention. Other QOL dimensions showed no statistically significant change. Qualitative findings reinforced the improvement in psychological and emotional well-being, with many participants describing a heightened sense of safety and peace of mind, often likening the technology to “insurance” or a silent companion. However, the impact on social relationships was paradoxical; some older adults felt more cared for, while others perceived a decline in in-person visits. This paradox highlighted the complexities of technology’s role in caregiving, as it might simultaneously enhance feelings of safety while unintentionally diminishing social connection for some older adults.

**Conclusions:**

AAL technologies such as Ubismart enhance older adults’ psychological and emotional well-being and sense of safety but may inadvertently reduce social interaction. Sustainable integration requires balancing these benefits with efforts to maintain meaningful caregiver connections, supporting both safety and social engagement for older adults.

**Trial Registration:**

ClinicalTrials.gov NCT06486935; https://clinicaltrials.gov/study/NCT06486935

## Introduction

### Background

“I am afraid of dying alone at home” [1]; this fear, expressed by an older woman in Singapore, underscores a growing global phenomenon. As health care advances have prolonged life expectancy [2], demographic projections suggest that by 2030, 1 in 6 people worldwide will be aged ≥60 years, thus entering older adulthood. Unfortunately, many factors, including declining birth rates, changing norms, urbanization, and migration, have led to the evolution of family structures, causing a considerable proportion of these older people to live alone. In the United States, about 28% of the people aged ≥65 years live by themselves, including slightly <6 million men and slightly >10 million women [3]. Similar trends are seen across the globe, such as in Canada [4], Singapore [5], and France [6]. While living alone as an older adult is not inherently problematic and can be an indicator of robust health [7], it remains challenging for some individuals, particularly impoverished older adults who rely on government or not-for-profit organizations’ support.

In response, developments in the Internet of Things (IoT) technologies and computational data science have introduced novel digital health interventions, such as ambient assisted living (AAL) technologies. AAL technologies are a type of smart home technology that enables individuals to remain socially connected while living independently [8]. Smart homes denote residences equipped with a high-technology network, linking sensors and other IoT-based domestic devices, appliances, and features that can be remotely monitored, accessed, or controlled and provide services that respond to the needs of the inhabitants [9]. The use of AAL technologies among older adults started to gain increasing research attention around the early 2000s [10,11]. These technologies hold an innovative promise in providing support for older adults, offering tools that enhance safety, independence, and overall quality of life (QOL) for those facing challenges associated with living alone [12,13].

However, end-user adoption and continued use of AAL technologies are still very low [8]. We believe that a significant obstacle to the sustainable use of AAL technologies lies in the absence of a robust, theory-driven approach to understanding how AAL technologies become routinely integrated and normalized in their social contexts. Intervention development and evaluation require a strong theoretical foundation [14]. Because social distancing protocols implemented during the COVID-19 pandemic have given impetus to an already growing trend in the adoption of AAL technologies [15,16], we believe that work theorizing the processes by which AAL technologies become routinely embedded in the care of older adults is both timely and relevant. Thus far, 3 distinct bodies of literature on AAL technologies have emerged: technical, human, and integrative perspectives [17]. The technical perspective primarily addresses design challenges and the implementation of AAL technologies. In contrast, the human perspective concerns the sociopsychological factors influencing adoption and shaping the use of AAL technologies.

While a great deal of good work has been done, recent research still highlights a lack of richer contextual and long-term approaches to understanding technology use among older adults requiring assisted living [18]. Indeed, most theoretical tools researchers use in the humanistic perspective of AAL technologies imply a psychological approach focusing on older adults as a unit of analysis. This is exemplified by the use of rational choice theories and theories of individual motivation, such as the theory of planned behavior, the technology acceptance model, and self-determination theory [19-21]. Such psychologizing of AAL technologies’ use greatly curtails an understanding of how sociopsychological factors shape the routinization of these technologies in the care of older adults. It neglects that technology acceptance in this context is culturally dependent and will vary from one society to another [18,22]. Indeed, older adults cannot be studied in isolation, especially those requiring assisted living, because they live in interdependence with one another, their caregivers, and technological interventions [17,18].

### Aims

Against this background, the aim of this paper is to promote the sustainable use of AAL technologies in the care of older adults by sharing a qualitative narrative and lessons learned from a longitudinal mixed methods study involving 19 older adults in Southeast Asia, a hitherto under-studied context. In doing so, we contribute to an emerging agenda of a rich sociological understanding of how older adults adopt and continue using AAL technologies by investigating an assemblage of sociotechnical linkages between different actors, including older adults, their caregivers, and the AAL technologies [18,23]. Such a focus is vital because AAL technologies can significantly impact the QOL for both older adults and caregivers if and only if they can be effectively integrated into routine practice [14].

To this end, we draw on 2 longitudinal AAL technology deployment studies to address the following research questions:

- How can AAL technologies be integrated and embedded into the care of older adults in context-dependent ways?
- What factors facilitate or impede the routine embedding of AAL technologies in the care of older adults?

In this paper, we first review related literature and introduce the theoretical framework guiding our narrative and proposed recommendations. Next, we detail the study’s context and the specific AAL technology implemented. Finally, we share key lessons from the technology deployment process.

### Prior Work

As mentioned earlier, a lot of great work has been done, and some researchers have also shared personal narratives and lessons learned. While these lessons learned by other researchers provide valuable insights, they lag in 3 important ways that motivate our work.

First, a few of these studies have used quantitative methods and found that several barriers, including usability concerns, a lack of perceived usefulness, and low technological efficacy, impede the routine integration of AAL technologies in older adults’ care [24-26], and indeed, these are helpful. However, richer contextual insights are difficult to capture through quantitative data [27]. Beyond their contributions to specific areas of empirical research, qualitative narratives can also serve to reorient theories. They may influence theories not only relating to individual AAL technology users but also regarding the society within which the use occurs. This is done by bringing attention to the social and cultural dynamics through which the routinization of these technologies occurs [28]. There are surely exceptions. Drawing insights from an ethnographic case study, Doyle et al [29] presented recommendations on the 4 interrelated and dynamic life cycles of independent living technology development: ethnographic inquiry, design, deployment, and ongoing evaluation. However, this differs from our objective to investigate sociopsychological and cultural dynamics that facilitate or impede the integration of AAL technologies into older adults’ care.

Second, some personal narratives lack an overarching theoretical framework to organize the key recommendations. For example, Consolvo et al [30] highlighted design insights supported by practical experiences, such as keeping the ambient sensors out of older adults’ view and providing a “human touch.” “Human touch” here refers to capturing qualitative details, such as the reasons behind specific actions or feelings, to create a more empathetic and personalized understanding of older adults’ experiences instead of focusing solely on sensor data. However, there is a lack of an overarching theory to create a complete and meaningful picture by linking their work to existing theories and previous research [31], which is understandable, as their work is design oriented and not psychosocial in nature. Still, to build a cumulative body of knowledge, personal narrative studies should be theoretically driven [32], as this will provide valuable support to the identification of patterns, themes, and connections in the narratives.

Third, only a handful of these studies were conducted in Southeast Asia. Most of those conducted in Southeast Asia used rational choice theories and quantitative approaches [9,33,34]. As indicated earlier, we believe qualitative research is more suited to understanding how AAL use evolves over time and why it evolves this way. The use of rational choice theories, with older adults being the primary data source, assumes that the decision to adopt and use a technology lies with the individual older user. However, the reality is far different because AAL technologies are better understood as complex adaptive systems, which are systems composed of interacting agents described in terms of rules [35]. Agents include the IoT components, older adults, and caregivers [35]. Such a view of AAL technologies’ use focuses attention on the fundamental mechanisms of emergence [36]; that is, the interactions between users and AAL technologies, along with their caregivers, are intricately linked and shape the overall context of AAL use [37]. Therefore, adoption, acceptance, and use are culture dependent and will vary from one society to another [18,22]. From such a perspective, the use of AAL technologies is viewed as (1) socially and culturally situated and (2) determined by the interactions of multiple actors, including older adults, their caregivers, and the AAL technologies themselves, who engage in nonlinear and, at times, chaotic ways [38].

### Organizing Theoretical Framework

It is recommended that personal narratives be shared within the confines of extant theoretical frameworks to better organize the lessons presented [32,38]. Therefore, we anchor our narrative and present our lessons based on normalization process theory (NPT) [14,39] because a fundamental theoretical and empirical puzzle in the complex health care interventions literature relates to how the use of these technologies can transcend conscious behavior and become part of normal routine in care activities [14,36]. NPT is a sociological theory explaining key mechanisms that facilitate or impede the implementation, embedding, and integration of new health techniques, technologies, and other complex interventions [39]. It is based on the premise that health care interventions are complex social interventions that require multiple people to work together to achieve a desired outcome [14]. It identifies 4 key mechanisms of the normalization process.

First, “coherence” pertains to target users’ sensemaking or understanding of the nature and purpose of the intervention. It is related to the notion of perceived usefulness in the information systems and human-computer interaction literature [40]. The intervention must make sense to the target users and be compatible with their values and expectations to be perceived as useful [41]. Second, “cognitive participation” means that the intervention must engage and include everyone at every health care system level, from patients and their families to health care professionals and administrators. Third, “collective action” means that the intervention needs to be integrated into routine practice and be supported by the necessary resources, infrastructure, and training. Finally, “reflexive monitoring” means that the intervention needs to be evaluated and monitored regularly to ensure that it remains effective and sustainable over time.

## Methods

### Ethical Considerations

This study received ethics approval from the institutional review board of the National University of Singapore (A-16-349; protocol title: “City for All Ages: Elderly friendly City Services for Active and Healthy Aging”) and is registered with ClinicalTrials.gov (NCT06486935). All participants were referred to the study by their caregivers. No reimbursement was offered for participation. Informed consent was obtained from each participant prior to enrollment, including consent for the future use of their data in research. All data used for analysis were deidentified to preserve participant confidentiality.

### Study Context

We situated our research within the context of older adults living alone in Singapore’s subsidized public housing, relying on professional caregiving services provided by government-supported care centers, specifically senior activity centers (SACs). Currently, SACs are expanding their reach and services under the newly adopted designation of active aging centers. The SACs provide various services, including social activities, monitoring homebound older adults, befriending them, and responding to emergency alerts. As with any other care facility, a daunting challenge for SACs is a shortage of workforce. Therefore, AAL technologies were found to be an innovative promise to help older adults age in place, which can be defined as remaining in the community, with some level of independence, rather than in residential care [42].

### Recruitment and Data Collection

#### Overview

Given the limited theoretical insights available, we adopted a mixed methods case study approach to explore the use of AAL technologies in this hitherto underexplored context. Our mixed methods approach consisted of semistructured interviews, a quantitative survey, and the AAL technology described in the subsequent sections.

We collected data at the individual level of analysis from 19 older adults and 2 caregivers. Drawing from our ethics review, the inclusion criteria for the older participants were (1) cognitively abled older adults living alone or with no more than 2 roommates; (2) English, Mandarin, or Cantonese speakers; and (3) members of the SAC. For ethical reasons, we excluded older adults with (1) severe disabilities, (2) reduced mobility (those who used a wheelchair), and (3) severe dementia. Older participants’ demographics are presented in Table 1, with most (10/19, 53%) participants in their 70s. Participants primarily spoke Mandarin or a combination of Mandarin and English, with varied educational backgrounds ranging from no formal education to secondary schooling. Most (16/19, 84%) were widowed and lived alone, although a few (3/19, 16%) resided with 1 or 2 family members.

**Table 1:** Demographic characteristics of older adults in this study.

ID	Sex	Age (y)	Language	Education	Marital status	Living arrangements
ID1	Female	65	Mandarin	None	Widowed	Alone
ID2	Female	70	English	Primary	Widowed	Alone
ID3	Female	74	Cantonese and Mandarin	None	Separated	Alone
ID4	Male	79	English	Primary	Married	Family
ID5	Female	86	Mandarin	Secondary	Single	Alone
ID6	Female	67	Mandarin and English	Primary	Widowed	Family
ID7	Female	67	Mandarin	None	Widowed	Alone
ID8	Male	72	Mandarin	Secondary	Married	Alone
ID9	Male	69	Mandarin and English	Secondary	Divorced	Alone
ID10	Female	70	English	Secondary	Widowed	Alone
ID11	Female	67	Mandarin and English	Secondary	Widowed	Alone
ID12	Female	71	Mandarin and English	Primary	Widowed	Alone
ID13	Female	70	Mandarin and English	Secondary	Widowed	Family
ID14	Female	90	Mandarin and English	None	Widowed	Alone
ID15	Female	70	Mandarin and English	None	Widowed	Alone
ID16	Male	83	Mandarin and English	Primary	Divorced	Alone
ID17	Male	77	Mandarin and English	None	Single	Alone
ID18	Female	73	Mandarin and English	Secondary	Widowed	Family
ID19	Female	82	Mandarin and English	None	Widowed	Alone

#### Quantitative Survey

Our primary dependent variable was older adults’ QOL. To assess this, we adopted the Older People’s Quality of Life Questionnaire (OPQOL) [43], which captures key dimensions of QOL relevant to older adult populations. Specifically, we focused on six core domains: (1) health, (2) independence and freedom, (3) psychological and emotional well-being, (4) social relationships, (5) home and neighborhood safety, and (6) leisure and social activities.

#### Semistructured Interviews

To complement and build on the quantitative findings, we conducted semistructured interviews with older adults. These interviews explored 3 key areas: Older adults’ perceived QOL, expectations of the AAL technology, and overall satisfaction with the AAL system following installation. First, perceived QOL was captured at baseline and throughout the study (Table 2). Perceived QOL included questions as follows: (1) How would you describe your QOL as a whole? (2) To what extent do you feel safe, given that you live alone? and (3) How is your relationship with the SAC caregivers? Questions related to expectations of the technology were as follows: (1) What are your expectations of this technology? (2) What do you think this technology can do for you? and (3) What would you like this technology to do for you? Questions related to satisfaction with the AAL technologies were as follows: (1) Overall, how satisfied are you with the sensor technology? (2) Would you recommend this technology to a friend or a relative? (3) What else would you like the technology to do for you? and (4) How has your relationship with the SAC caregivers changed since the technology implementation?

**Table 2:** Ubiquitous Service Management and Reasoning Architecture deployment details.

Region	Duration (mo)	Starting date	Homes, n	Sensors
Geylang Bahru, Singapore	1	August 2016	3	×10^a^
Geylang Bahru, Singapore	>6	March 2017	5	×10
Geylang Bahru, Singapore	>6	March 2017	5	Z-Wave
Ang Mo Kio, Singapore	>9	May 2018	6	Z-Wave

^a^10 sensors deployed throughout the 2 homes.

We included caregivers who were full-time staff members at the SAC and fluent in English, Mandarin, or Cantonese. Both caregivers were male, held the position of senior executive at the SAC, and were actively involved in caregiving responsibilities across 2 centers.

Our questions for the caregivers covered aspects of their job and how AAL technologies could help (ie, gauging expectations at baseline) and have helped (ie, gauging experiences following deployment and the older adults’ perceptions of the technology). Examples of questions are as follows: (1) What aspects of your job do you like, and what aspects of your job do you find fulfilling? (2) What prospective improvements do you wish to see in your work? (3) What technologies are currently available to you in your daily work? (5) How do you think sensor-based technology can facilitate your work? (6) How do you think sensor-based technology can hinder your work? (7) To your understanding, what are some of the older adults’ concerns pertaining to technology? and (8) What were some of the reasons for some individuals not providing consent to participate? All interviews were recorded and transcribed.

### The AAL Technology Intervention

#### Overview

We developed an AAL technology platform termed Ubiquitous Service Management and Reasoning architecture (Ubismart), a live web-based platform with multiple interfaces and communication channels that are continuously expanded [44,45]. The platform has been deployed in real homes in France and Singapore. In this paper, we focused on Singapore deployments, which occurred in 3 waves with incremental features in 2016, 2017, and 2018 (Table 2).

The entire process that was organized using the NPT spanned, on average, 14 months, from ethics approvals and development to reflexive monitoring (Table 3).

**Table 3:** Global timeline from user needs to deployments.

Months	Activities	
	NPT^a^ phase	Descriptions
1-5	Development and approvals	Observations, discussions, and prototyping Validation and demo Applications for ethics approval Initial trial and field test
5	Coherence	Briefing sessions
6	Cognitive participation	Recruitment sessions and baseline interviews
7	Collective action	AAL^b^ intervention deployment
8-12	Reflexive monitoring	Feature updates and maintenance Follow-up interviews Data analysis and feedback
13-14	Feedback on AAL normalization	Interviews and impact on validation

^a^NPT: normalization process theory.

^b^AAL: ambient assisted living.

**Figure 1:**
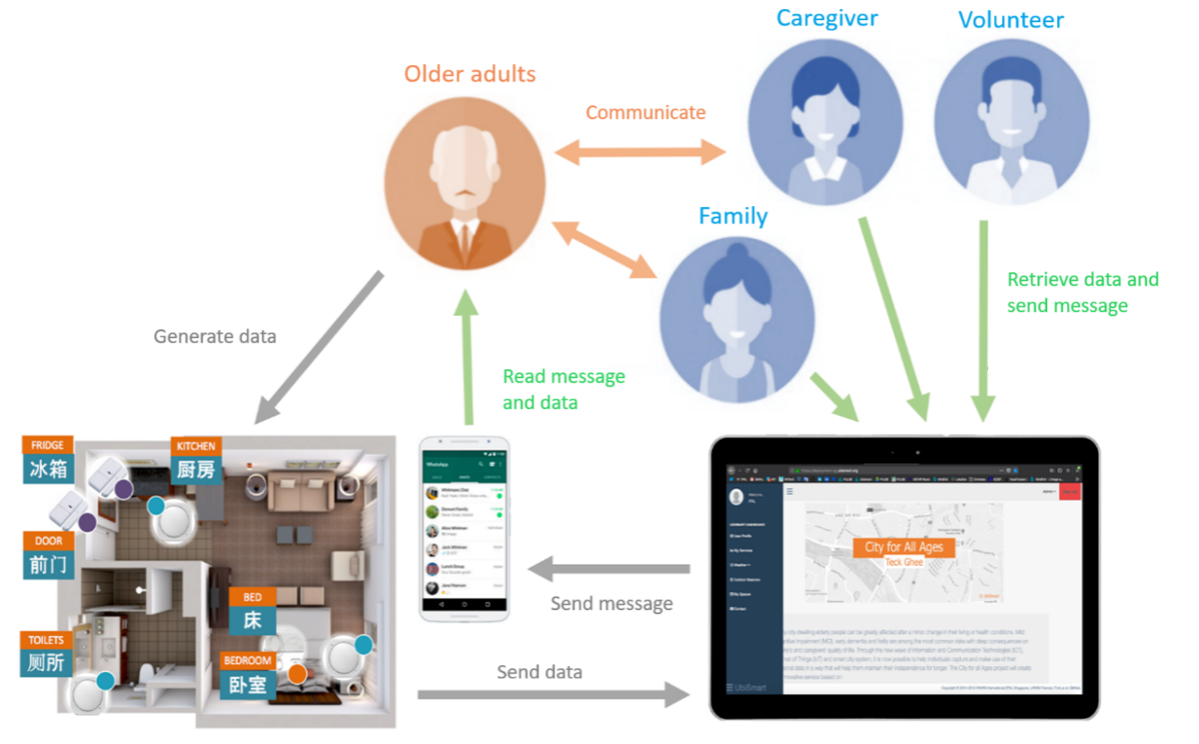
Overview of the Ubiquitous Service Management and Reasoning Architecture setup and stakeholder interactions.

We subsequently present the components of the Ubismart system depicted in Figure 1. The system unobtrusively monitored older adults’ activities of daily living (ADLs) and generated data through sensors placed in key areas of their homes. These data were then accessible to caregivers through a connected platform, allowing for remote communication, data retrieval, and message exchange to support the older adults’ well-being. Ubismart consisted of 3 key modules, discussed in the subsequent sections.

#### Module 1: Multimodal Sensors

**Figure 2:**
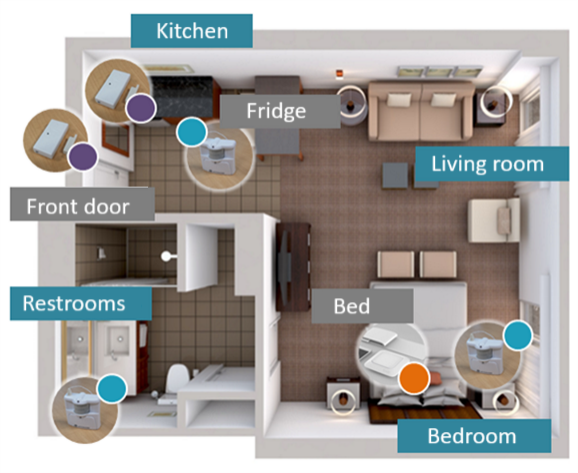
Deployment layout in older adults’ apartments.

Multimodal sensors with wireless data transmission capability were installed at different locations (eg, bedroom, kitchen, toilet, bathroom, and living room) to monitor and detect the activities performed by individual older adults, such as cooking, sleeping, going to the bathroom, going out of the apartment, etc. As illustrated in Figure 2, we installed (1) contact sensors on the fridge and house doors; (2) motion sensors in the kitchen, bedroom, and toilet; and (3) bed sensors under the mattress.

**Figure 3:**
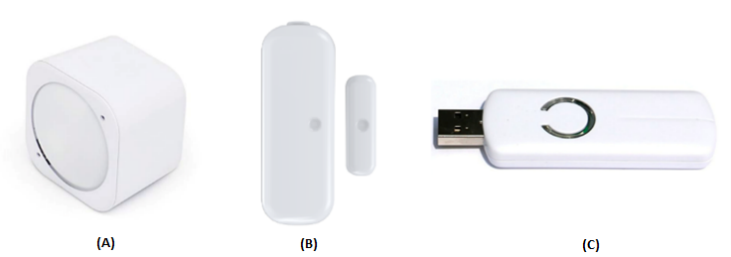
Z-Wave equipment—multisensor (eg, motion and humidity temperature), contact sensor, and Z-Wave receiver (from left to right).

Contact sensors capture when doors are opened and closed [46]. Motion sensors emit a signal when there is a significant change in the infrared spectrum; therefore, they are used to detect movement (Figure 3). The technical specifications of the sensors are discussed elsewhere [47].

**Figure 4:**
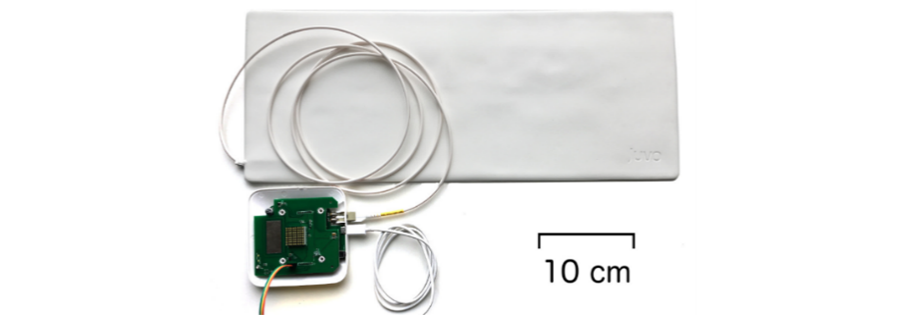
Sleep mat from JUVO.

The microbend fiberoptic pressure sensor mat, developed by Juvo Labs Pte Ltd (Figure 4), was placed unobtrusively below the bed mattress or sheet to measure heart rate and respiratory rate during sleep. This mat provides information on sleep parameters, such as sleep-wake rhythms, and can be used to detect sleep disorders. Technical specifications of this mat and related algorithm development are published elsewhere [47-50].

**Figure 5:**
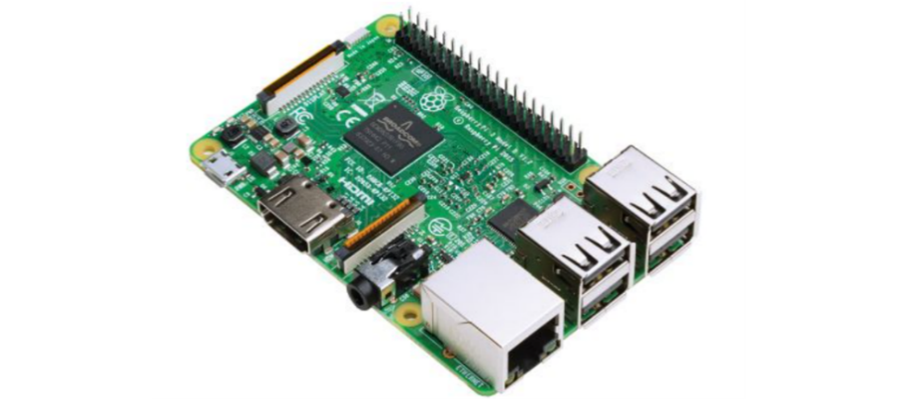
Raspberry Pi 3 Model B.

The collected data were transferred through a secure gateway with a Raspberry Pi (Raspberry Pi Foundation) to a dedicated server for data processing and analysis. A Raspberry Pi is a pocket-sized computer commonly used in IoT applications because of its small size and ability to run Unix-like systems that ease the portability of algorithms (Figure 5). Our installation was strictly devoid of any video and audio processing or recording to respect the privacy of our participants.

#### Module 2: Bluetooth Beacons

**Figure 6:**
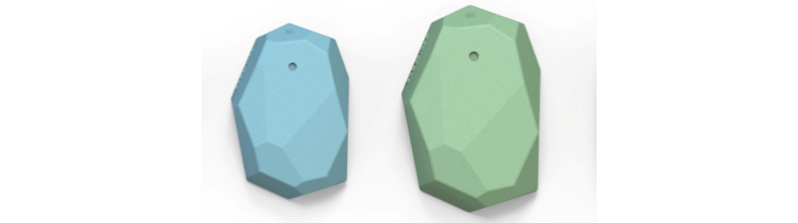
Bluetooth beacons.

We strategically placed Bluetooth beacons from Estimote (Figure 6) in public areas to track the engagement of older adults in outdoor events organized by the SAC. These activities included gardening sessions, bingo games, and various social gatherings. It is important to note that the beacons do not receive or process any data, and they are not aware of any detections. They are placed at specific locations, and at regular intervals, they emit their own identifiers. The tracking is performed using a custom-made smartphone app that detects these signals and sends data to our servers.

#### Module 3: Mobile App

**Figure 7:**
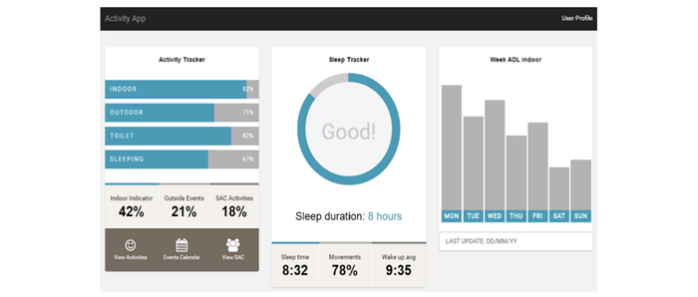
Screenshot of an older adult’s activities of daily living in Ubiquitous Service Management and Reasoning Architecture.

**Figure 8:**
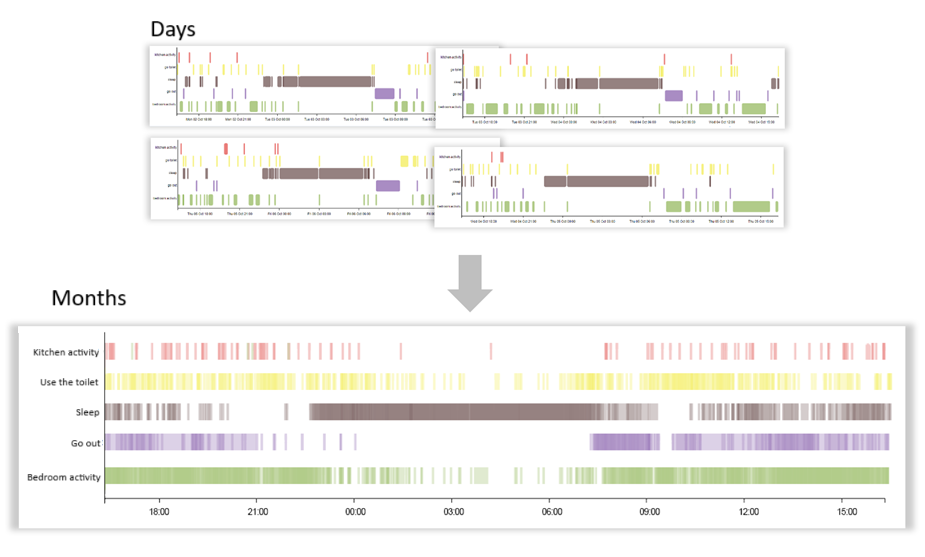
Personalized processing to detect deviations from older adults’ baseline data.

The data from the sensors were processed in real time to provide a visualization of older adults’ activities on a mobile app, as schematized in Figures 7 and 8. This app was accessible to the local staff at the SAC and the adults’ relatives (if applicable). The goal of the app was to provide information on older adults’ QOL in a cognitively efficient manner. As shown in Figures 7 and 8, visualized data included sleep duration, relative duration spent indoors and outdoors, and washroom and kitchen activities [51,52]. Caregivers received notifications of any irregularities (ie, deviations from the established baseline) through the mobile app. The baseline ADLs for the older adults were established through comprehensive semistructured interviews before deployment (Table 3). Risk alerts were activated when there were indications of significant and concerning behavioral changes. These could be detected through various parameters, including reduced participation in social events (as recorded by SAC data); noticeable deterioration in performing basic ADLs, including mobility or challenges in preparing meals during regular hours; and a decrease in sleep quality or sleep-related concerns.

### Shortcomings of Previous AAL Technologies Versus This Study’s Approach

Having described the Ubismart system, we turn to how it deviates from systems in previous literature. As mentioned, the number of studies on AAL technologies started to soar around 2015 [53], meaning that our deployment was one of the first studies to use AAL technologies in the care of older adults, with our first deployment in August 2016 (Table 2). Previous and current AAL technology deployments made use of audio or visual input. This is an increasingly pressing issue that our deployments avoided by excluding these sources of information. Privacy and fear of recordings are important aspects during deployments. From a technical perspective, we realized the concept of a “smart home in a box” by developing a system that can be deployed in any environment within 30 minutes. This was achieved through efforts to streamline sensor registration and create an easily extensible system built on semantic web principles [54,55]. In addition, our proprietary algorithms enabled noninvasive sleep tracking and could detect potential respiratory issues [56].

## Results

### Quantitative Survey: the Impact of Ubismart on QOL

As outlined earlier, we had initially planned to assess QOL using the validated OPQOL instrument. However, pilot testing revealed significant difficulties. Many participants, especially those with no formal education (7/19, 37%) or only primary schooling (6/19, 32%), struggled to meaningfully differentiate between points on the Likert scale, even when the survey was translated into local dialects. These challenges posed a risk of introducing measurement error and participant disengagement.

**Figure 9:**
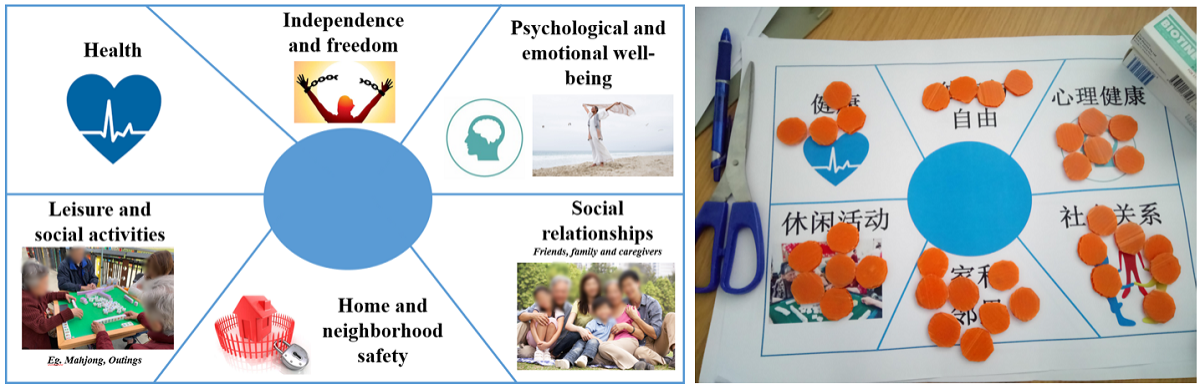
The gamified quality of life measurement tool.

To ensure comprehension and more meaningful self-expression, we developed a gamified version of the QOL assessment (Figure 9). Participants were given 35 coins and asked to allocate those across 6 core QOL dimensions. The root question was simplified to “How do you feel you are doing in each of these areas?” The number of coins assigned to each domain served as an intuitive proxy for participants’ self-assessed well-being in that area.

We selected 35 coins to strike a balance between simplicity and sensitivity. The number was large enough to allow participants to express meaningful variation across the 6 domains while still being cognitively manageable for older adults with limited numeracy.

Using data collected through the gamified QOL assessment tool, we conducted a paired sample, 2-tailed *t* tests to examine whether the installation of AAL technology led to significant changes in the distribution of perceived well-being across QOL domains. Because the total number of coins (n=35) was fixed at all time points, our focus was not on changes in overall QOL scores but rather on how participants reallocated their perceived well-being across individual dimensions. Because the assessment was simplified to the domain level, we interpreted any meaningful shifts in specific domains as indicative of potential changes in participants’ overall QOL.

When we analyzed QOL across its individual dimensions, 2 areas showed statistically significant gains. For leisure and social activities, participants reported a significant increase in scores following the intervention (before: mean 2.67, SD 2.29; after: mean 4.67, SD 1.22; *P*=.03), indicating enhanced social engagement and participation. Similarly, psychological and emotional well-being improved significantly (before: mean 5.44, SD 1.51; after: mean 6.78, SD 1.48; *P*=.01), suggesting a positive impact on emotional stability and mental wellness.

No statistically significant changes were observed in the dimensions of health, independence and freedom, social relationships, or home and neighborhood safety (all *P*>.05). The absence of change in “independence and freedom” was expected, given that the system was designed to be unobtrusive and did not alter participants’ physical mobility or routines. Similarly, “health” was largely outside the scope of the AAL system, which did not provide direct medical interventions. However, the lack of significant changes in “social relationships” and “home and neighborhood safety” was unexpected. These findings are further explored in the A Qualitative Narrative of the Impact of Ubismart on Older Adults’ QOL section, which offers deeper insight into how participants perceived and experienced these domains beyond what was captured through quantitative measures.

### A Qualitative Narrative of the Impact of Ubismart on Older Adults’ QOL

#### Overview

Triangulating findings from the quantitative analysis, our semistructured interviews likewise did not reveal any perceived impact on health and independence and freedom. As mentioned earlier, this was not unexpected. First, participants might have attributed their health status primarily to age-related conditions, and the system itself did not offer medical functions or interventions. In addition, no major health events occurred during the 14-month study period that would have activated the system’s monitoring features. Second, participants continued to age in place, and the unobtrusive nature of the intervention meant their autonomy and routines remained unchanged, factors that likely contributed to a stable sense of independence and freedom.

In the subsequent sections, we have presented qualitative findings for the remaining 4 QOL dimensions: emotional well-being, safety, leisure and social activities, and social relationships. We highlighted how participants described the intervention’s perceived impact in each area.

#### Home and Neighborhood Safety

Safety emerged as one of the most frequently discussed and emotionally resonant themes in the qualitative narratives, even though it did not register as statistically significant in the quantitative analysis. Participants consistently described a heightened sense of home-based safety, citing reassurance during emergencies, confidence in being monitored, and peace of mind while sleeping alone.

While the quantitative instrument combined home and neighborhood safety into a single construct, participants’ narratives overwhelmingly emphasized in-home safety, suggesting that this specificity may have been masked in the statistical findings. These qualitative accounts highlighted that, even without measurable shifts in coin allocations, participants experienced the intervention as increasing their sense of security within their living spaces. The following quotes illustrate these perceptions:

I feel safer...

Participant 2

I feel that it [Ubismart] is very good for its sense of safety. If anything happens to me, [the caregivers] would know...If nothing is happening for the time being, then all is good...While we sleep at night, we feel safer.

Participant 10

#### Psychological and Emotional Well-Being

The deep-seated fear of dying or struggling alone, reflected in the Introduction section [1] and echoed by many older adults in our study, was a central theme in participants’ narratives about psychological and emotional well-being. While the AAL system was not designed to provide emotional care, its unobtrusive presence appeared to ease anxieties and offer a sense of psychological reassurance. Indeed, both quantitative and qualitative data pointed to improvements in psychological and emotional well-being, making this one of the strongest areas of alignment across methods. Participants frequently described feeling calmer, more secure, and less fearful since the installation of Ubismart. The system was perceived not just as a technological safeguard but as a “silent companion” that offered comfort in the absence of continuous human presence. The following excerpts illustrate this enhanced psychological and emotional state:

Without your [the team’s] help, my troubles will still persist. As [a senior], I have so much to be fearful of. But with these sensors, I feel so much at ease. I need not fear anything. It’s better than not having the sensors...It’s like having someone by my side...Without the sensors, there’s nothing else I have; who else other than God can I ask?

Participant 5

It’s psychology. You feel more confident, you feel more secure. It’s the psychology.

Participant 6

It is like having insurance for us. If anything happens to us, the [caregivers] will know, for example, if we have a fall. So, the sense of safety is very good...It is like my personal insurance, which is very good for me.

Participant 7

These individual accounts were echoed by caregivers, who also observed shifts in the participants’ sense of security:

They feel that there is a heightened sense of security, meaning that even when they are at home, they have a better peace of mind with this technology.

Caregiver 1

#### Leisure and Social Activities

Quantitative analysis showed a statistically significant improvement in leisure and social activities, suggesting increased participation or engagement following the intervention. However, this domain was not particularly salient in older adults’ qualitative narratives. When probed, most participants did not mention any changes in their social routines or activity levels that they attributed to Ubismart. To better understand this discrepancy, we consulted the caregivers, who offered a potential explanation:

...the whole deployment doesn’t really affect their sense of independence and [SAC activities]. Those who before this research project are regular participants continue to be regular participants for the SAC’s activities or community events. Those who are mostly home-bound continue to stay home-bound.

Caregiver 2

#### Social Relationships

Surprisingly, our findings revealed a mixed impact on the domain of social relationships. While some participants described strengthened bonds with caregivers following the deployment of Ubismart, others expressed feelings of being overlooked or less visible. One older adult noted that caregivers had become more attentive:

They have become more caring, clearer, and more understanding.

Participant 10

In contrast, another participant conveyed a sense of isolation:

I just hope that those downstairs [the caregivers] will look up to the old people more often. I am lonely in this flat and should have someone come up to visit me, right...Here is [senior] who cannot conveniently move her hands and legs, and yet there is no one to visit.

Participant 5

These divergent perspectives reflected the nuanced reality of digitally supported care in a low-income, high-need setting. In this study, most (16/19, 84%) older adults lived alone in subsidized rental housing and had limited or no regular contact with close family. For many, SAC caregivers represented their most consistent and trusted source of support not only for physical well-being but also for emotional connection and social presence. In such a context, even subtle shifts in caregiver engagement were felt acutely.

It is important to note the clear differences among these older adults. Participant 10 was relatively frail, while participant 5 was healthier and more independent. According to the caregiver, standard protocol required visiting older adults who needed more support 3 times a week, while healthier older adults received 2 visits per month. The caregiver further explained that if the Ubismart app showed no anomalies and the older adults remained active at the SAC, they sometimes forwent additional visits due to limited resources:

So, [participant 5] is not exactly frail and vulnerable, so it is a subjective comment, but I should say that sometimes this thing about home visits is because [participant 5] and [participant 10] are the more regular participants in the center. They come to the center on a weekly basis, at least three or four times. So, as long as the center gets to see them, interact with them, and understand whether they are okay or good for that day or that week. That is also a form of outreach.

While some older adults interpreted Ubismart as a sign of care, others perceived it as a potential substitute for relational presence, a perception that risks reinforcing feelings of abandonment if not accompanied by continued in-person interaction. However, caregivers also highlighted that Ubismart facilitated deeper relational exchanges. The mobile app served not only as a monitoring tool but also as a conversational catalyst:

We have a more in-depth understanding of the [older person’s] daily condition. We will use [the app] as a conversational starter so that we will get into a more in-depth interaction with them and be more able to care for particular characteristics.

In fact, the technology was especially welcomed in light of staffing limitations. One caregiver explained the following:

So currently, we have five [full-time caregivers] as per headcount, so it’s [divided with the other] SAC...so it’s just two staff per center. Our latest estimate is slightly more than 800 [older people]; about 400 belong to the rental units, and the rest are all from the nearby blocks.

Caregiver 1

Against this backdrop, caregivers appreciated Ubismart’s ability to extend their reach while maintaining dignity and privacy for older adults:

We are very supportive of this because we feel this approach is nonintrusive. It respects the seniors’ privacy...and we are able to ensure that they are okay at home.

Caregiver 2

These reflections reinforced the view that AAL technologies, when thoughtfully implemented, could serve as a bridge rather than a barrier to meaningful human connection, especially in contexts where caregiving capacity is stretched thin.

### Concerns of the AAL Technologies

Although our primary focus was on QOL, we also explored participants’ broader experiences with the Ubismart system, including any concerns that arose during its use. As with many technological interventions, older adults expressed several reservations. Through open coding of the interview data, we identified 5 key concerns. The first related to electricity consumption, which was raised both during interviews with the research team and in conversations with SAC caregivers:

I asked them, “Why [is] my electric bill very high?” [The caregiver said] “No, you got a sensor, but this one is like a battery.”

Participant 2

I think one of the most common [questions] is whether it consumes a lot of electricity. But right now, based on the 19 participants, they are aware that these things don’t take much electricity.

Caregiver 2

Second, privacy violation was a major concern in the beginning. The older adults suspected that the sensors might have video recording capabilities. Some took afternoon naps on their chairs instead of the bed out of fear that the bed sensor would capture it, and they might be judged for unproductivity. However, we explained to the older adults that the goal is to keep them safe and not judge their sleep patterns. In addition, privacy concerns waned over time as the older adults understood that the sensors did not have video recording capabilities. This is reflected in the following quote:

I was initially concerned about the sensors taking videos in the toilet. But later I got used to it.

Participant 6

Third, physical harm was a major concern for one of the frail participants. She suspected that the sensors were the cause of her body pain:

I’m scared [that] maybe the [sensors] are affecting my whole legs...now my legs cannot go up, [it’s] very painful

Participant 2

As a result, she wanted to uninstall the sensors. After reassurance from her nurse that the pain was not attributable to the sensors and that the sensors would be beneficial in case of an emergency, the participant chose to continue using the technology:

I asked if it’s better for them [the team] to take out [the sensors], but the nurses said, “No, no need, no need because it is better for you.” They’re scared [if anything happens] when I go to the toilet and fall down or sleep and cannot wake.

Fourth, two participants had concerns about the inconveniences caused by technology in some ADLs, such as cleaning:

I personally don’t like the sensors under the bed. Every time I clean under the bed, it drops, and I do not know how to put it back. If I lay it on the floor, it looks untidy as well.

Participant 3

[It is already difficult] to clean the floor. Last time, I could clean. But with the sensors, I cannot [clean].

Participant 2

After a closer look at the sensors, we found that the bed sensor required an extension cable, which we informed the older adults could be elevated to not obstruct the cleaning. This resolved the issue. Once the aforementioned concerns were addressed, most of the participants were satisfied with the system. They seemed happy to have something new in their home. We believe that they felt special to have been chosen to participate in this study because the older adults consistently asked if the sensors could be left at their homes after the study had ended.

Fifth, fears about radiation exposure unexpectedly emerged as a key barrier to participation. Although we had addressed more common concerns, such as electricity consumption, privacy, and cleaning inconveniences, our initial planning did not anticipate that some participants might associate ambient sensors with radiation risk. During the initial briefing, one older adult who appeared to be an informal leader among peers voiced this concern and declined to proceed with installation. His decision significantly influenced others, some of whom had previously agreed to participate, leading to a noticeable drop in participation.

### Lessons Learned and Recommendations

#### Overview

The subsequent sections present lessons learned at different phases of the AAL technologies’ integration and normalization process. Before outlining specific insights, we reiterate the distinctiveness of the study context to make it salient for interpreting the recommendations mentioned subsequently. As previously described, our participants were older adults living in heavily subsidized rental housing in Singapore. Many (16/19, 84%) lived alone, had little or no regular contact with close family, and depended heavily on staff at the SACs as their primary source of emotional and social support. In this context, caregiving relationships were not merely transactional. They were deeply relational and, for many participants, irreplaceable. This high-need, low-resource environment shaped how the Ubismart intervention was introduced, understood, and integrated into daily life. The recommendations presented in this section are thus grounded in and should be interpreted through this unique caregiving and social ecosystem.

#### Coherence

Coherence refers to how individuals and collectives make sense of a new intervention, that is, what it is, why it matters, and how it fits into their lives [14,39]. In this study, fostering coherence required more than technical explanation; it demanded sensitivity to the lived realities of older adults who relied heavily on SAC caregivers for trust, translation, and emotional anchoring. Recognizing that caregivers played a pivotal role in participants’ day-to-day lives, we engaged SAC staff members early and invited them to colead participant briefings. Their presence not only facilitated translation because many (16/19, 84%) older adults communicated in Hokkien, Mandarin, or Cantonese but also lent credibility to the project. Because SAC caregivers were often viewed as family-like figures, their involvement helped older adults feel reassured and respected during the introduction of Ubismart. On the basis of these insights, we present some recommendations.

The first recommendation is to engage trusted caregiving intermediaries early and include them in participant briefings, as their involvement plays a critical role in fostering coherence by translating, legitimizing, and contextualizing the intervention.

The second recommendation is to deliver briefings in linguistically and culturally appropriate formats, such as using translated materials, visual aids, and caregiver-facilitated discussions, to ensure understanding among older adults with diverse language abilities and educational backgrounds.

#### Cognitive Engagement

Cognitive engagement focuses on individuals’ active participation in the intervention [14,39]. Routine integration of AAL technologies into older adults’ care hinges on their willingness to actively engage in field deployment and commitment to maintaining the presence of ambient sensors within their homes. In our context, we initially assumed that older adults’ trust in SAC caregivers, coupled with a clear understanding of the AAL system’s benefits, would translate into widespread acceptance. However, our experience revealed that this assumption overlooked the layered dynamics of peer influence and perceived risk, which proved critical to engagement.

Despite initial enthusiasm, a subset of participants opted out of deployment due to fears surrounding radiation exposure, a concern introduced by one particularly influential older adult. This individual’s withdrawal prompted others to follow suit. Notably, this occurred even after the system’s safety and privacy features were explained. While the Ubismart system used only low-power, off-the-shelf electronics, technical reassurance alone was insufficient to counteract socially transmitted anxieties. This experience highlighted that engagement depended not only on formal information channels but also on informal social dynamics. Surfacing and addressing these perceived risks (however unfounded) required relational attentiveness and not just technical accuracy. Drawing from this, we make further recommendations.

The third recommendation is to identify and engage both formal (eg, SAC caregivers) and informal (eg, respected peers at the SAC) influencers early in the process. For older adults who spend much of their time in communal settings, peer relationships significantly shape perceptions and decisions. Addressing perceived risks requires not only technical transparency but also relational attentiveness and messaging that resonates with these trusted figures.

The fourth recommendation is to provide ample time for reflection on the proposed intervention. Although we asked the older adults about their concerns at the joint briefing, we may not have given them sufficient time to reflect. Therefore, it would be best to allow older adults sufficient time (eg, a few days) for reflection, encouraging them to document their apprehensions for a thorough concern mitigation process.

The fifth recommendation is to facilitate low-pressure, small-group discussions at SACs where participants can share thoughts with peers and caregivers. These informal settings encourage open dialogue and can serve as early warning systems for surfacing emerging concerns or misinformation. They also provide a trusted space for gently debunking myths, such as health risks from sensors, through respectful, peer-sensitive conversation.

#### Collective Action

Collective action addresses how stakeholders within an ecosystem work together to enact the new intervention [14,39]. In the context of this study, successful deployment of Ubismart required not only technical infrastructure but also thoughtful collaboration between older adults and SAC caregivers to ensure the system was meaningfully integrated into everyday life. While our team gleaned many general insights about sensor placement, connectivity, and troubleshooting (eg, SIM card recharging, battery drain, and optimal sensor placement), we focused on lessons uniquely shaped by our study context—a dense, low-income housing estate in Singapore where older adults often lived alone, had limited family interaction, and relied heavily on SAC caregivers for daily support.

A critical insight that emerged during early deployment was the influence of “saving face,” a deeply rooted cultural value in many Asian societies. Face is the social esteem accorded by others or the respectability or deference that a person can claim for themselves from others by the degree to which they are judged to have behaved appropriately [57]. To avoid losing face, one participant reported napping on a chair rather than on the bed so her rest would not be captured by the bed sensor. She feared that being seen as inactive during the day might reflect poorly on her. The technology itself did not drive this concern but a broader social anxiety around appearing unproductive or burdensome did.

This behavior underscored a central challenge in the era of big data, namely data accuracy. Sensor-based systems like Ubismart generate large volumes of behavioral data that, if interpreted without cultural context, can misrepresent reality. Inaccuracies, especially those stemming from altered behavior due to social pressure, pose a serious threat to the quality and utility of big data in AAL systems. Ensuring data integrity requires building environments where users feel safe to act naturally. To support this, clear and culturally sensitive messaging is essential. Repeatedly emphasizing that the system is not intended to evaluate or penalize routine behaviors but instead to detect deviations that may signal distress can prevent compensatory behaviors that compromise data fidelity. Therefore, we make some recommendations mentioned subsequently.

The sixth recommendation is to reinforce early and often that the goal of the AAL system is to detect anomalies and not to surveil or judge daily behavior. This framing supports cultural values like dignity and reduces compensatory behaviors that compromise data quality.

The seventh recommendation is to leverage peer-led and caregiver-facilitated discussions at SACs to build trust and clarify how sensor data are used. These relational channels help reduce fear, promote natural behavior, and improve data reliability in a context where both peer influence and caregiver relationships are central.

Along the same vein of data accuracy, one of the core challenges in big data systems like AAL is maintaining reliable sensor performance over time. In our context, many (16/19, 84%) older adults lived alone and lacked the technical know-how to detect or report sensor issues, while SAC caregivers were already operating at full capacity. This made routine manual checks unfeasible. However, regular interactions with peers and caregivers at the SACs present a culturally grounded opportunity. By integrating informal sensor checks into existing social routines, such as weekly visits or group activities, we can support data reliability without adding strain to caregiving staff. Therefore, we make the recommendation mentioned subsequently.

The eighth recommendation is to leverage trusted peer and caregiver networks to identify sensor issues. SAC caregivers and socially influential peers should be equipped with simple cues to detect malfunctions, and informal checks during regular interactions should be encouraged. These efforts should be paired with automated alerts for a hybrid approach to reliability.

#### Reflexive Monitoring

This phase of the NPT is concerned with how users will perceive the intervention once it has been in use for a while [14,39]. In our study, both older adults and SAC caregivers perceived Ubismart as a helpful, dignified addition to daily routines. Caregivers reported that the system facilitated more personalized and proactive care, while older adults described a growing sense of safety, particularly within the home environment. This perceived security appeared to catalyze subtle behavioral improvements, including increased confidence and reduced anxiety, aligning with the statistically significant gains observed in psychological and emotional well-being.

However, the same system also revealed unintended consequences. As the technology assumed a more active monitoring role, some participants perceived a decline in relational contact. While caregivers relied on Ubismart to allocate limited resources and prioritize high-need cases, this shift risked reinforcing feelings of neglect or abandonment among older adults who previously received more frequent visits. In the context of this study, where most (16/19, 84%) participants lived alone and SAC caregivers served as their main source of emotional support, the reduction in in-person interactions carried emotional weight.

These dynamics underscored a core tension in digitally supported care; while ambient technologies might improve efficiency and safety, they could inadvertently diminish the very human connections older adults value most. Therefore, reflexive monitoring requires ongoing attentiveness not only to technical metrics but also to lived experiences and shifting care relationships. Drawing from the aforementioned lessons, we present two recommendations.

The ninth recommendation is to embed structured opportunities for older adults and caregivers to share how technology affects their care experiences and relationships. These feedback mechanisms not only surface unintended consequences (eg, reduced visit frequency) but also provide a relational check on system use, reinforcing dignity, trust, and care continuity.

The 10th recommendation is that in high-need, low-resource settings, where caregivers are the primary source of support, AAL technologies should augment relational care and not replace it. To address capacity gaps, caregivers should partner with community volunteers, students, or nonprofits to ensure older adults’ emotional and social needs are met.

## Discussion

### Principal Findings

Motivated by the shortage of longitudinal studies on the integration of AAL technologies in older adults’ care, especially in Southeast Asia, we used an in-depth case study to explore the deployment and impact of an AAL technology, which we termed Ubismart, on older adults’ QOL. Our study revealed that AAL technologies positively impacted certain QOL dimensions for older adults, particularly enhancing psychological well-being and a sense of security. Many participants reported feeling safer with the technology, describing it as a reassuring presence in their homes. However, the impact on social relationships was mixed; while some older adults felt more cared for due to the technology, others experienced fewer in-person visits and a sense of isolation, as caregivers increasingly relied on AAL systems for monitoring.

Key lessons emerged regarding the integration and normalization of AAL technology. First, to establish coherence, it is essential to prioritize culturally grounded and relationally sensitive strategies that help older adults understand the purpose and relevance of AAL technology. Involving caregivers (ie, trusted figures in participants’ lives) in joint briefings would be especially effective in building trust and reinforcing the system’s value within existing care relationships. Second, to support cognitive engagement, it is crucial to recognize that adoption is shaped not only by formal caregivers but also by influential peer networks within the ecosystem. In our study, a single participant’s concern about radiation led others to withdraw, demonstrating how quickly skepticism can spread in tightly connected communities. Third, collective action requires sensitivity to cultural norms such as saving face. Some participants altered their behavior, such as napping on a chair, to avoid perceived judgment. Reinforcing that the system monitors for anomalies, not habits, is key to preserving data accuracy. Finally, reflexive monitoring brought attention to an important unintended consequence; while the technology promoted safety, it also inadvertently reduced social interactions for some older adults, highlighting the need for vigilance in balancing AAL technology’s benefits with ongoing human connection.

### Comparison With Prior Work

As mentioned earlier, much good work has been done on the use of AAL in the care of older adults. Similar to other projects, including HomeAssist [58] and NESTORE [59], both conducted in the European Union, our intervention supported aging in place by offering unobtrusive monitoring. Overall, our findings align with previous AAL studies that reported improved QOL among older adults [60,61]. However, our work contributes novel insights into several key areas.

First, as Taramasco et al [60] mentioned, data pertaining to QOL are scarce. They highlighted the methodological challenge of having to adjust for baseline imbalances in QOL measures before detecting a significant effect, underscoring how sensitive and complex this construct can be to capture. Our study contributes to this conversation not only by examining QOL outcomes but also by reflecting critically on the measurement process itself. We document the challenges of using standard Likert-based tools with older adults who have limited formal education and propose a culturally adapted, gamified alternative that offers a more accessible way for participants to express their perceived well-being. This approach enables us to surface meaningful shifts in specific QOL domains while also contributing to a broader methodological discussion on how best to measure QOL in underserved populations.

Second, recent works have shown that about 5 countries (ie, the United States, the United Kingdom, France, Canada, and Japan) have been leading research in AAL technologies thus far, and of these 5, only Japan has a collectivistic culture [62]. Answering the call for more research from diverse cultures [62], our study offers rare empirical insights from a subpopulation within a collectivistic culture. Specifically, we focused on older adults in Singapore who, despite living in a society that values familial interdependence, often experience social isolation due to impoverishment, limited education, and estrangement from close family. These older adults reside in heavily subsidized housing and depend primarily on caregiving staff at community-based SACs for emotional and social support. Unlike the typical AAL studies conducted in individualistic, high-resource settings, our work surfaces how the adoption and routinization of AAL technologies are mediated by unique cultural and relational dynamics, including peer influence, saving face [57], and the central role of institutional caregivers. These findings highlight the importance of designing AAL systems not only for technical robustness but also for cultural fit and relational integration within the lived caregiving ecologies of susceptible older adults.

Third, the OPQOL is one of the most widely used and validated instruments for assessing QOL in older adult populations, offering a multidimensional perspective that includes domains such as health, independence, social relationships, emotional well-being, and home and neighborhood safety [43,63]. However, our findings suggest that this latter domain, particularly in both the full [43] and brief versions [63] of OPQOL, may mask intervention-specific effects when home and neighborhood safety are treated as a single construct. Although our quantitative analysis using a gamified proxy of the OPQOL did not show a statistically significant change in the aggregated safety domain, qualitative narratives consistently revealed an increased sense of home-based safety. Participants described feeling reassured knowing help would be available during emergencies, especially at night or when living alone. In contrast, neighborhood safety was rarely mentioned and remained largely unaffected by the intervention. While the OPQOL-brief includes the item “I feel safe where I live,” this phrasing still conflates 2 distinct contexts. On the basis of our findings, we suggest that future studies consider explicitly separating this domain into “I feel safe at home” and “I feel safe in my neighborhood” to more accurately detect the localized effects of AAL interventions and avoid measurement dilution.

Taken together, these contributions reinforce the need for culturally sensitive, relationally grounded, and methodologically flexible approaches to designing and evaluating AAL technologies, particularly in diverse, underrepresented populations.

### Limitations

As with all research, this study is not without its limitations. We highlight 3 limitations that we believe are particularly important. First, a potential limitation of this study is that we focused on one context, Singapore. While the findings of our study may be generalizable to similar cultures (ie, collectivistic), some of our findings, especially pertaining to the relationship between older adults and caregivers, may not be applicable in individualistic cultures. However, our goal was to explore a context-specific understanding of the routinization of AAL technologies in this hitherto under-studied context. In doing so, we answer calls for research that redirects focus from general theories of technology use to context-specific theorizing [64]. Second, even though we explored our study context comprehensively, by having 19 older adults in this study, we are again unable to claim broad generalizability of the proposed recommendations. More research is needed before such claims can be made. However, we note that our sample size is comparable to similar studies with sample sizes ranging from 9 to 20 [24,29], owing to the cost of deployment. Third, we used a validated measure of QOL that is largely used in Western contexts [43]; therefore, our participants struggled to understand it, and we had to improvise and create the “gamified QOL measurement tool” as a conversation starter. Indeed, QOL may be experienced differently based on demographic differences [65].

### Future Directions

Future research should address the aforementioned limitations to enhance the study’s robustness and applicability. First, cross-cultural studies that examine AAL technology use in both collectivistic and individualistic contexts could provide insights into the role of cultural values in shaping older adults’ and caregivers’ dynamics. This would help determine the extent to which our findings apply beyond Singapore. Second, while our sample size aligns with similar studies, larger and more diverse samples would strengthen the generalizability of our recommendations. Future studies could partner with health care organizations to increase sample size and diversity. Finally, developing culturally responsive QOL measurement tools would mitigate the challenges we encountered with Western-centric QOL assessments, allowing a more accurate understanding of well-being across diverse populations.

### Conclusions

In conclusion, this study highlights the promise of AAL technologies in addressing the challenges faced by older adults living independently; however, it underscores that no technology is a panacea. While AAL tools can enhance safety, social connection, and QOL, they cannot fully substitute for the support systems that older adults need. Future research should continue to explore these technologies’ potential across different cultural contexts and emphasize the importance of complementary social and familial support in promoting older adults’ well-being.

## Abbreviations

AAL: ambient assisted living
ADL: activity of daily living
IoT: Internet of Things
NPT: normalization process theory
OPQOL: Older People’s Quality of Life Questionnaire
QOL: quality of life
SAC: senior activity center
Ubismart: Ubiquitous Service Management and Reasoning Architecture
